# Supplementary material for: WordCommentsAnalyzer: A windows software tool for qualitative research
Source: F1000Res. 2018 Sep 4;7:536. Originally published 2018 May 3. [Version 2] doi: 10.12688/f1000research.14819.2 (PMC6137411; doi:10.12688/f1000research.14819.2)
Supplement: Supplementary file 3 [file f1000research-7-17614-s0002.tgz › b2a79496-4ada-47c4-9682-a99ce867836d.zip!/Eight Iranian Health Journals/Journal of Knowledge and Health_3.docx]

© Journal of Knowledge and Health. This work is licensed under CC BY 4.0 (https://creativecommons.org/licenses/by/4.0/).

Volume & Issue: دوره 12، شماره 3: 1396

Title: اثر تمرینات تناوبی شدید بر سطوح آپلین بافت چربی زیرپوستی و احشایی، انسولین و گلوکز پلاسمایی در موش‌های صحرایی نر

http://knh.shmu.ac.ir/index.php/site/article/view/1674

مقدمه: در سال‌های اخیر، شیوع چاقی همه‌گیر شده است و فعالیت ورزشی قوی‌ترین محرک برای کاهش آن است. امروزه بافت چربی به‌عنوان یک آدیپوکاین شناخته شده است که تعداد زیادی سایتوکاین و هورمون ترشح می‌کند. هدف از تحقیق حاضر بررسی اثرات 5 هفته تمرینات تناوبی شدید (HIIT: High intensity interval training) بر سطوح آپلین در بافت چربی زیرپوستی و احشایی و میزان انسولین و گلوکز خون در موش‌های نر ویستار است. مواد و روش‌ها: 20 سر موش به‌طور تصادفي به دو گروه تمرین (HIIT) و گروه کنترل تقسيم شدند. کل برنامه تمرینی به مدت پنج هفته و با پنج جلسه در هفته انجام شد. آزمودنی‌ها در هفته اول با سرعت  m/min37 در 6 وهله دو دقیقه‌ای فعالیت کردند و در بین هر وهله نيز به مدت یک دقیقه به آنها استراحت داده شد. در هفته دوم تعداد وهله‌ها به 7 مرحله رسید و سرعت به m/min40 افزایش یافت. در هفته سوم، پروتکل تمرینی با سرعتی برابر با  m/min43 و در 9 وهله انجام شد. در هفته چهارم سرعت به m/min 49 و تعداد وهله ها به 10 و در پنجمین هفته تعداد وهله‌ها به 12 و سرعت به  m/m52 رسيد. 72 ساعت پس از آخرين جلسه تمريني، نمونه‌برداري بافت چربی احشایی و زیرپوستی و خونگیری انجام و متغیرهای موردنظر اندازه‌گیری شد. برای تحلیل داده‌ها نیز از آزمون تي مستقل استفاده شد. سطح معناداري، 05/0 در نظر گرفته شد. نتایج: نتايج، تغییرات معني‌داری را در سطوح آپلین چربی احشایی و چربی زیر پوستی و انسولین پلاسماي گروه تمرين نسبت‌به گروه كنترل نشان داد (05/0P<) اما این تغییرات در گلوکز پلاسما معني‌دار نبود (05/0P≥). نتیجه‌گیری: نتایج حاصل از این تحقیق نشان داد که تمرینات HIIT مي‌تواند باعث کاهش سطوح آپلین در بافت چربی احشایی و زیر پوستی ‌شود که این عامل مي‌تواند از بروز بیماری‌های مرتبط با چاقی جلوگیری کند. همچنین کاهش آپلین مي‌تواند کاهش مقاومت به انسولین و افزایش حساسیت انسولینی را به همراه داشته باشد.

Title: تأثير تمرینات اینتروال شدید برآسیب ناشی از ایسکمی- ریپرفیوژن میوکارد در رت‌های نر ویستار

http://knh.shmu.ac.ir/index.php/site/article/view/1702

مقدمه: پیش‌‌شرطی‌سازی ایسکمی، اثرات حفاظتی در مقابل آسیب ناشی از ایسکمی- ریپرفیوژن میوکارد فراهم می‌کند. تمرینات ورزشی حالتی از پیش‌شرطی‌سازی ایسکمیک است كه از طریق کاهش فشار اکسیداتیو می‌تواند موجب کاهش اندازه ناحیه انفارکتوس پس از ایسکمی ریپرفیوژن گردد. هدف از این مطالعه بررسی اثرات حفاظتی تمرینات اینتروال با شدت بالا بر فشار اکسایشی و آسیب ناشی از ایسکمی- ریپرفیوژن میوکارد در رت‌های نر نژاد ویستار بود. مواد و روش‌ها: ۲۰ رت نر ویستار به‌طور تصادفی در دو گروه کنترل (10n=) و گروه تمرینات اینتروال شدید (10n=)  قرار داده شدند. موش‌های گروه تمرینی به مدت هشت هفته تمرینات اینتروال شدید (۵ جلسه در هفته، هر جلسه به مدت ۳۰ دقیقه) را انجام دادند. تمرینات اینتروال شدید شامل 10 مرحله فعالیت دویدن دو دقیقه‌ای با شدت 90 درصد حداکثر اکسیژن مصرفی و یک دقیقه دویدن با شدت ۵۰ درصد حداکثر اکسیژن مصرفی بود. گروه کنترل در این مدت هیچ‌گونه فعالیتی نداشتند. ۷۲ ساعت پس از آخرین جلسه تمرینی، حیوانات هر دو گروه با انسداد شریان پایین‌رونده قدامی میوکارد، تحت جراحی قرار گرفته و در شرایط ایسکمی (۳۰ دقیقه) ریپرفیوژن (۱۲۰ دقیقه) قرار گرفتند. پس از اتمام ایسکمی- رپرفیوژن، مالون‌دی‌آلدئید (MDA)، فعالیت آنزیم‌های گلوتاتیون پراکسیداز (GPX)، کاتالاز (CAT) و میلوپراکسیداز (MPO) و اندازه ناحیه انفارکتوس میوکارد اندازه‌گیری شد. نتايج: نتایج نشان داد که ميزان فعالیت آنزیم گلوتاتیون پراکسیداز و میلوپراکسیداز متعاقب آسیب ناشی از ایسکمی- ریپرفیوژن بین گروه اینتروال شدید و گروه کنترل، تفاوت معناداری نداشت (05/0≤P). باوجود این، فعالیت آنزیم کاتالاز و محتوی گلوتاتیون میوکارد (004/0=P) در گروه تمرینات اینتروال به‌طور معناداری نسبت‌به گروه کنترل بیشتر بود. همچنین مشاهده شد كه غلظت مالون دی‌آلدئید در گروه تمرینی به‌طور معناداری نسبت‌به گروه کنترل بیشتر بود (006/0=P). علاوه بر اينها، اندازه ناحیه انفارکتوس نيز در گروه تمرینی نسبت‌به گروه کنترل کمتر بود (004/0=P). نتیجه‌گیری: در نهایت می‌توان گفت که تمرینات اینتروال با شدت بالا با ایجاد شرایط پیش‌شرطی‌سازی ایسکمیک از طریق افزایش برخی از شاخص‌های آنتی‌اکسیدانی، اندازه ناحیه انفارکتوس ناشی از ایسکمی- ریپرفیوژن میوکارد را کاهش می‌دهد و می‌تواند به‌عنوان یک روش مهم پیشگیرانه جهت جلوگیری از اتفاقات ایسکمیک میوکارد مورد توجه قرار گیرد.

Title: نقش عوامل مشتق از سلول‌های اندوتلیال در بروز کوه‌گرفتگی حاد

http://knh.shmu.ac.ir/index.php/site/article/view/1733

مقدمه: کوه گرفتگی حاد که ناشی از صعود سریع به ارتفاعات می‌باشد دارای شرایط پاتوفیزیولوژیکی پیچیده است. بنابراین، در این مطالعه به بررسی نقش ‌عامل‌های مشتق از سلول‌های اندوتلیال در بروز کوه گرفتگی حاد پرداخته شده است. مواد و روش‌ها: ۲۱ فرد سالم با میانگین سنی 5/8±7/31 سال در این مطالعه شرکت کردند. نمونه‌گیری خون به‌صورت ناشتا در سطح دریا، 1 و ۲۴ ساعت بعد از صعود، از سیاهرگ بازویی آزمودنی‌ها گرفته شد و سپس میزان VEGF و اندوتلین-۱ به‌روش الایزا مورد اندازه‌گیری قرار گرفت. همچنین، میزان کوه گرفتگی حاد توسط پرسشنامه لیک لوئیس در ۶ ساعت پس از در معرض قرارگرفتن به ارتفاع مورد ارزیابی قرار گرفت. نتایج: ۱۱ نفر از آزمودنی‌ها در ۶ ساعت پس از قرار گرفتن در معرض ارتفاع، دچار کوه‌گرفتگی حاد شدند. میزان VEGF و اندوتلین-۱ در سطح دریا در افراد مقاوم به AMS نسبت‌به افراد حساس به AMS بیشتر بود (05/0P≤). میزان VEGF و اندوتلین-۱ در گروه مقاوم و حساس به AMS در ۱ و ۲۴ ساعت پس از قرارگرفتن در معرض ارتفاع به‌طور معناداری افزایش پیدا کرد (05/0P≤). همچنين میزان VEGF و اندوتلین-۱، در حالت پایه و در ۱ و ۲۴ ساعت پس از قرارگرفتن در معرض ارتفاع در افراد مقاوم به AMS نسبت‌به افراد حساس به AMS بیشتر بود (05/0P≤). نتیجه‌گیری: نتايج مطالعه نشان داد كه پاسخ این پروتئین‌ها پس از قرارگرفتن در معرض ارتفاع در افراد مقاوم به AMS نسبت‌به افراد حساس به AMS بیشتر است و حساسیت به AMS ممکن است با پاسخ کمتر VEGF و اندوتلین-۱ مرتبط باشد.

Title: ارزيابي كيفيت پساب تصفيه‌خانه فاضلاب اكباتان جهت استفاده مجدد در آبياري كشاورزي با استفاده از مدل CWQI

http://knh.shmu.ac.ir/index.php/site/article/view/1759

مقدمه: هدف این مطالعه، كاربرد مدل (Canadian water quality index) CWQI براي ارزيابي كيفيت پساب تصفيه‌خانه فاضلاب اكباتان جهت استفاده مجدد از آن در آبياري كشاورزي بود. مواد و روش‌ها: این مطالعه از نوع مطالعه توصیفی- تحلیلی بوده و به مدت 48 ماه بر روی پساب خروجی از تصفیه‌خانه فاضلاب اكباتان انجام شد. به‌منظور تعیین کیفیت پساب جهت استفاده در بخش کشاورزی با استفاده از شاخص CWQI، مقدار 22 شاخص‌ فیزیکوشیمیایی و 3 شاخص‌ میکروبی در طی چهار فصل و به‌صورت هفته‌اي يك نمونه (و در مجموع 54 نمونه) اندازه‌گيري شد. نتايج حاصله سپس وارد برنامه CWQI Calculator گرديد تا تناسب كيفيت پساب اين تصفيه‌خانه براي آبياري كشاورزي مورد بررسي قرار گيرد. نتایج: مقدار CWQI پساب اين تصفيه‌خانه براي آبياري كشاورزي برابر با 56 به‌دست آمد كه به مفهوم فاصله‌داشتن کیفیت پساب از سطح مطلوب خود مي‌باشد. براساس نتايج برنامه، مقدار CWQI براي آشاميدن، پرورش آبزيان و تغذيه دام و طيور نيز به‌ترتيب برابر با 30، 38 و 53 به‌دست آمد. همچنین بهترین کاربرد اين پساب باتوجه به مقدار CWQI، تغذيه دام و طيور می‌باشد. نتیجه‌گیری: نتایج مطالعه نشان داد که کیفیت پساب این تصفیه‌خانه برای آبیاری کشاورزی مناسب است ولی در حد کیفیت مرزی قرار دارد. مهمترین عوامل پایین بودن مقدار CWQI، كدورت و جيوه مي‌باشند. باتوجه به نتایج مطالعه، مي‌توان از پساب اين تصفیه‌خانه برای آبياري كشاورزي استفاده كرد و از به‌كارگيري آب‌هاي زيرزميني باكيفيت خودداري كرد.

Title: مقایسه‌ی اثر یک دوره تمرینات هوازی به همراه تمرینات پیلاتس بر روی هموستاز گلوکزخون دانش‌آموزان دختر چاق و با وزن طبیعی

http://knh.shmu.ac.ir/index.php/site/article/view/1666

مقدمه: چاقی، به‌عنوان یک مشکل اساسی ناشی از رفتار خوردن و شرایط متابولیک بدن مطرح است که ارتباط مستقیمي ‌با هموستاز گلوکز دارد. هدف اصلی این تحقیق مقایسه‌‌ اثریک دوره تمرینات هوازی به همراه تمرینات پیلاتس بر مقادیر انسولین پلاسما، قندخون ناشتا و مقاومت به انسولین در دختران چاق و با وزن طبیعی بود. مواد و روش‌ها: جامعه آماری تحقیق، دانش‌آموزان یکی از دبیرستان‌های منتخب شهر لاهیجان بود. در این مطالعه‌‌ نیمه تجربی، 15 دختر نوجوان سالم چاق و 15 نفر با وزن طبیعی به‌طور داوطلبانه شرکت کردند. نمونه‌ها به‌صورت نمونه‌گیری در دسترس انتخاب شدند. برنامه تمرین شامل تمرینات هوازی و پیلاتس به مدت 6 هفته، 3 جلسه در هفته و هر جلسه 90 دقیقه با شدت 65 تا 80% ضربان قلب هدف بود. متغیرهای هموستاز گلوکز قبل و بعد از پایان برنامه‌‌ تمرینی اندازه‌‌گیری شد. تحلیل آماری به کمک نرم‌‌افزار SPSS و در سطح معنی‌‌داری 05/0≥P انجام شد. داده‌های جمع‌‌آوری شده با استفاده از آماره‌هایt  وابسته و مستقل مورد تجزیه و تحلیل قرار گرفت. نتایج: میانگین سن در دو گروه چاق و با وزن طبیعی به‌ترتیب 7/0±6/13 و 6/0± 8/13 سال و شاخص توده‌‌ بدني آنها به‌ترتيب، 8/1±8/28 و 2/2±2/20 کیلوگرم بر متر مربع بود. نتایج تحقیق نشان داد که 6 هفته تمرین همزمان هوازی و پیلاتس به‌طور معنی‌‌داری مقادیر وزن (004/0P=) و شاخص توده بدنی (001/0P=) و میزان انسولین و مدل هموستاز مقاومت به انسولین (01/0P=) را در دختران چاق بیشتر از دختران با وزن طبیعی کاهش مي‌دهد. نتیجه‌گیری: به‌نظر مي‌‌رسد 6 هفته تمرین همزمان هوازی و پیلاتس مي‌‌تواند موجب کاهش بیشتر وزن و شاخص توده بدنی در دختران چاق و نیز کاهش معنی‌دار در میزان انسولین و مدل هموستاز مقاومت به انسولین در دختران چاق در مقایسه با دختران با وزن طبیعی گردد.

Title: بررسي آسیب به شبکیه چشم متعاقب مصرف سیکلوسپورین A

http://knh.shmu.ac.ir/index.php/site/article/view/1731

مقدمه: داروی سیکلوسپورین A به‌عنوان یک داروی مهارکننده سیستم ایمنی در بسیاری از بیماری‌ها تجویز می‌گردد. از جمله عوارض ناشی از مصرف سیکلوسپورین، اختلالات بینایی و آسیب به شبکیه است. روش‌های متفاوتی جهت بررسی آسیب به شبکیه موجود است که از بهترین آنها تکنیک الکترورتینوگرام است. هدف مطالعه حاضر، بررسی اثرات مصرف سیکلوسپورین بر شبکیه بیماران مبتلا‌به آرتریت روماتوئید بود. مواد و روش‌ها: این مطالعه مشاهده‌ای که به‌صورت توصیفی- تحلیلی انجام شد ۵5 بیمار مصرف‌کننده سیکلوسپورین به‌علت آرتریت روماتوئید در سال 1394-1395 به‌صورت در دسترس انتخاب شده و تحت آزمون الکترورتینوگرافی قرار گرفتند و ولتاژ و فاز تأخیری در آنها تعیین و با گروه طبیعی مقایسه شد. نتایج براساس میانگین، گزارش و با استفاده از آزمون تجزيه و تحليل واریانس تجزیه و تحلیل شد. سطح معنی‌داری نيز 05/0 در نظر گرفته شد. نتایج: میانگین ولتاژ ثبت شده در ERG برابر با 7/74 با انحراف معیار 7/29 بود که اختلاف آماری معناداری را با مقدار طبيعي یعنی 125 نشان می‌داد (0001/0=P). میانگین فاز تأخیری ثبت شده نيز ۹/3۳ با انحراف معیار ۵8/۳ بود که اختلاف آماری معناداری را با مقدار طبيعي یعنی 3۹/۳۲ نشان نمی‌داد (545/0=P). نتیجه‌گیری: براساس نتایج حاصل از این مطالعه چنین استنباط می‌شود که مصرف سیکلوسپورین سبب کاهش معناداری در ولتاژ ثبت شده در الکترورتینوگرام می‌شود اما بر روی فاز تأخیری مؤثر نمی‌باشد. لذا بیماران مصرف‌کننده این دارو باید تحت نظر چشم پزشک روند درمانی خود را ادامه دهند.

Title: استفاده از فرآیند فنتون برای کاهش حجم لجن بیولوژیکی صنایع لبنی

http://knh.shmu.ac.ir/index.php/site/article/view/1777

مقدمه: لجن فعال يكي از فرآيندهاي متعارف تصفيه بيولوزيكي فاضلاب در تصفيه‌خانه‌هاي فاضلاب صنعتي مي‌باشد. در اين فرآيند، مقدارزيادي لجن توليد مي‌شود كه هزينه تصفيه و دفع آن، بخش عمده‌اي از هزينه‌هاي تصفيه فاضلاب را به خود اختصاص مي‌دهد. هدف اين مطالعه، بررسي اثر فرآيند فنتون بر كاهش حجم لجن صنايع لبني بود. مواد و روش‌ها: این مطالعه، از نوع مطالعات تجربی است که در آن به تعیین اثر فرآیند اکسیداسیون شیمیایی با پراکسید هیدروژن و فنتون بر کاهش حجم لجن صنایع لبنی پرداخته شده است. براي اين منظور تأثیر شاخص‌های pH (1، 2، 3، 4 و ...10)، نسبت [Fe2+]/[H2O2] (5/0 و 1 و 5/1 و 2)، زمان ماند (2 تا 12) و غلظت پراکسید هیدروژن (500 و 1000 و 1500 و 2000 و 2500) مورد بررسی قرار گرفت. کلیه آزمایش‌ها مطابق روش‌هاي ذكر شده در كتاب استاندارد متد (2004) انجام گردید. نتايج: براساس نتايج مطالعه، حداکثر ميزان حذفVSS  در زمان تماس 6 ساعت، pH برابر با 3، نسبتFe2+]/[H2O2]  برابر با 1 و غلظت پراکسید هیدروژن برابر با 1500 و به میزان 82 درصد حاصل شد. براساس نتايج به‌دست آمده، در صورت نبود یون Fe2+ در محيط، كارامدي سیستم برابر صفر بود و بیشترین درصد حذفVSS  نيز به ميزان 82 درصد در نسبت[Fe2+]/[H2O2]  برابر با 1 مشاهده شد. همچنين براساس نتايج به‌دست آمده، با افزايش غلظت پراكسيد هيدروژن، مقدارVSS  كاهش يافت. نتیجه‌گیری: نتايج مطالعه نشان داد كه فرآيند فنتون، گزينه‌اي ممكن و كارآمد براي كاهش حجم لجن صنايع لبني مي‌باشد و به‌كارگيري آن سبب بهبود كيفيت لجن، بهينه‌سازي فرآيند تثبيت و تسهيل نمودن شرايط بهره‌برداري مي‌شود.

Title: تأثير همزمان فعالیت تمرینی منظم و ویتامین D بر سطوح NF-κBp65 در رت‌های نر قرار گرفته در معرض آب اکسیژنه

http://knh.shmu.ac.ir/index.php/site/article/view/1762

مقدمه: استرس اکسیداتیو و التهاب نقش محوری در عملکرد قلب و اختلالات آن بازی می‌کنند و در مسیرهای سیگنالی مشترکی سهیم هستند. زیر واحد p65 عامل هسته‌ای کاپا B (NF-κBp65)، از نشانگران مهم التهاب میوکارد است. هدف پژوهش حاضر، بررسی تأثير همزمان فعالیت تمرینی منظم (E)، ویتامین  D(VD) و القای استرس اکسیداتیو بر سطوح NF-κBp65 در میوکارد می‌باشد. مواد و روش‌ها: 60 سر رت نر بالغ نژاد ویستار به‌طور تصادفی به 10 گروه (6=n) شامل: گروه شاهد، دریافت آب اکسیژنه (H2O2)، دریافت آب اکسیژنه دو برابر (2H2O2)؛ H2O2 +VD؛ 2H2O2+VD؛ H2O2+E؛ 2H2O2 +E؛ H2O2+VD+E؛ 2H2O2+VD+E و دی‌متیل سولفوکساید+سالین تقسیم و به مدت 8 هفته تحت پروتکل مداخله قرار گرفتند. سپس غلظت پروتئین NF-κBp65 با کیت الایزا اندازه‌گیری و با استفاده از تحزيه و تحليل واریانس سه طرفه تحلیل شد. سطح معناداري برابر 05/0 در نظر گرفته شد. نتایج: یافته‌ها نشان داد كه تمرین باعث افزايش غلظت پروتئین NF-κBp65 شد (049/0=P). تأثير غلظت H2O2 نيز بر افزايش غلظت پروتئین NF-κBp65 معنادار بود (013/0=P). همچنين تعامل تمرین و VD اثر کاهنده بر غلظت پروتئین NF-κBp65 داشت (004/0=P). با این حال، اثر VD و تعامل تمرین با H2O2 بر غلظت پروتئین NF-κBp65 معنی‌دار نشد. همزمانی سه مداخله تمرین، VD و H2O2 نسبت‌به جمع اثر هریک، اثری کاهنده بر غلظت پروتئین NF-κBp65 داشت (038/0=P).نتيجه‌گيري: به‌نظر می‌رسد که می‌توان از القای برو‌‌ن‌زاد استرس اکسیداتیو، فعالیت تمرینی منظم و مصرف VD جهت پیش‌آماده‌سازی و کاهش التهاب میوکارد در شرایط بالابودن استرس اکسیداتیو استفاده نمود.

Title: تأثیر استعمال سیگار بر روی توانایی‌های سایکوموتور رانندگی: سرعت درک و هماهنگی دو دستی

http://knh.shmu.ac.ir/index.php/site/article/view/1779

مقدمه: مطالعات اپیدمیولوژیک نشان می‌دهد كه سیگاری‌ها بیشتر در معرض خطر حوادث ترافیکی هستند. هدف از این مطالعه بررسی تأثیر استعمال سیگار بر روی توانایی مشاهده‌ای یا سرعت درک و هماهنگی دست- چشم و دست - دست از توانایی‌های سایکوموتور رانندگی است. مواد و روش‌ها: این مطالعه از نوع نیمه تجربی بوده که در سال 1396 در آزمایشگاه مرکز تحقیقات تروما و پژوهش‌های جراحی سینا وابسته‌به دانشگاه علوم پزشکی تهران انجام شد و در آن 22 نفر دانشجوی مرد 20 تا40 ساله دانشگاه‌های تهران که به‌طور گاهگاهی سیگار می‌کشیدند به‌طور داوطلبانه شرکت کردند. ابزار جمع‌آوری اطلاعات شامل 2 آزمون از بسته روانشناسی ترافیک سیستم کامپیوتری وینا (VTS)(Vienna test system) و دو پرسشنامه وابستگی به نیکوتین (فاگرستروم) و مشخصات فردی و دیدگاه آنها در مورد سیگار بود. تجزیه و تحلیل اطلاعات با استفاده از روش مدل آمیخته (mixed model) انجام شد. نتایج: میانگین± انحراف معیار سن و نمره وابستگی به نیکوتین (فاگرستروم) شرکت‌کنندگان در مطالعه به‌ترتیب 3 ± 6/25 سال و 4/1 ± 1/1 به‌دست آمد و 5/45 درصد از شركت‌كنندگان ابراز داشتند که استعمال سیگار، رانندگی آنان را کمی بد یا بدتر می‌کند اما نتايج مطالعه، ارتباط آماری معنی‌داری بين توانایی مشاهده‌ای یا سرعت درک و هماهنگی دست- چشم و دست - دست با استعمال سیگار را نشان نداد (05/0≤P). نتیجه‌گیری: نتایج مطالعه نشان داد که استعمال یک نخ سیگار در افرادی که گاهگاهی سیگار می‌کشند تغییر آماری معنی‌داری در هماهنگی و سرعت درک که از توانایی‌های سایکوموتور رانندگی مورد نیاز برای رانندگی ایمن است ایجاد نمی‌کند. به‌منظور تعيين تمامي جنبه‌هاي تأثير سيگار بر توانايي‌هاي سایکوموتور رانندگی، انجام مطالعه بر روی سیگاری‌های دائمی با انواع نمره وابستگی به نیکوتین پیشنهاد می‌گردد.
